# Sheehan’s syndrome presenting with panhypopituitarism and central diabetes insipidus: a case report

**DOI:** 10.1186/s12902-024-01654-w

**Published:** 2024-07-23

**Authors:** Chin-Fang Chen, Yu-Cheng Liang, Meng-Jie Tsai, Horng-Yih Ou

**Affiliations:** Department of Internal Medicine, College of Medicine, National Cheng Kung University Hospital, National Cheng Kung University, No. 1 University Road, Tainan, 70101 Taiwan

**Keywords:** Adrenal insufficiency, Central Diabetes insipidus, Panhypopituitarism, Postpartum hemorrhagic shock, Sheehan’s syndrome

## Abstract

**Background:**

Sheehan’s syndrome is a rare condition, which is classically characterized by anterior pituitary hypofunction following postpartum shock or hemorrhage. While diabetes insipidus (DI) is not commonly associated with Sheehan’s syndrome, we present a rare case of a multiparous female developing rapid-onset panhypopituitarism and DI following severe postpartum hemorrhage.

**Case presentation:**

A previously healthy 39-year-old woman, gravida 5, para 4, presented with hypovolemic shock after vaginal delivery, attributed to severe postpartum hemorrhage, necessitating emergent hysterectomy. Although her shock episodes resolved during hospitalization, she developed intermittent fever, later diagnosed as adrenal insufficiency. Administration of hydrocortisone effectively resolved the fever. However, she subsequently developed diabetes insipidus. Diagnosis of Sheehan’s syndrome with central diabetes insipidus was confirmed through functional hormonal tests and MRI findings. Treatment consisted of hormone replacement therapy, with persistent panhypopituitarism noted during a ten-year follow-up period.

**Conclusions:**

Sheehan’s syndrome is a rare complication of postpartum hemorrhage. Central diabetes insipidus should be suspected, although not commonly, while the patient presented polyuria and polydipsia. Besides, the potential necessity for long-term hormonal replacement therapy should be considered.

## Background

Sheehan’s syndrome is a rare condition, which is classically characterized by anterior pituitary hypofunction following postpartum shock or hemorrhage. While diabetes insipidus (DI), which instead involves the posterior pituitary peptide hormone, is not commonly associated with Sheehan’s syndrome [1, 2], we present a case of a multiparous female who experienced a unique combination of rapid onset of panhypopituitarism and DI following a episode of severe postpartum hemorrhage.

## Case presentation

A previously healthy 39-year-old woman, gravida 5, para 4, at 41 weeks pregnant, was transferred to our emergency department in hypovolemic shock. Three hours earlier, she had delivered a healthy full-term male infant weighing 3500 g through vaginal delivery, without the use of any instruments or excessive uterine pressure. However, one hour after delivery, significant postpartum vaginal bleeding occurred, despite receiving intravenous Oxytocin (40IU, IV) and Methylergonovine (0.8 mg, IV). Consequently, she received a transfusion of 10 units of packed red blood cells (RBC) and 1 unit of fresh frozen plasma (FFP). The estimated blood loss was 3,000 ml before transferring to our emergency department.

Upon arrival, the patient was presented with profound shock, including a body temperature of 34.8 °C, respiratory rate of 30 breaths per minute, and undetectable blood pressure. Physical examination revealed stupor consciousness, pale conjunctiva, and severe respiratory distress. Initial laboratory findings showed hemoglobin 10.0 g/dL, platelet 113,000 cells/mm3. Arterial blood gas analysis indicated pH 6.601, PCO_2_ 104.9 mmHg, bicarbonate 10.4 mmol/L, and PO_2_ 30 mmHg. Coagulation studies indicated prothrombin time 18.75/11.10 s and an activated partial thromboplastin time 185.55/28.9 s. Due to her critical condition, an emergent hysterectomy was performed, revealing a laceration of the right uterine artery, with a substantial transfusion of 9 units of whole blood, 37 units of packed RBC, 36 units of platelets, and 36 units of FFP, due to intraoperative blood loss of 11,300 ml. Following the surgery, the patient was admitted to the intensive care unit.

The patient’s hospitalization became complicated when she developed catheter-related sepsis and the bloodstream infection caused by *Enterococcus faecalis* and *oxacillin-resistant Staphylococcus sciuri*. Additionally, she experienced disseminated intravascular coagulopathy, acute renal insufficiency, and ischemic hepatitis. Proper administration of fluids and antibiotics, including vancomycin, ampicillin and sulbactam, led to improvements in renal and liver function and the recovery from the shock episode. Despite completing a course of antibiotics, the patient experienced intermittent fever for 2–3 weeks. Subsequently, she was diagnosed with adrenal insufficiency based on low cortisol levels (8 AM/4 PM: 5.6/4.0 µg/dL). The fever resolved after administering intravenous hydrocortisone 100 mg daily. However, she soon developed polyuria on the 30th day, with a significant increase in urine output, up to 11,070 ml/day. Given the clinical suspicion of Sheehan’s syndrome with DI, further investigations were conducted.

To assess anterior pituitary function, the patient underwent Insulin hypoglycemia/ thyrotropin-releasing hormone (TRH) test/ luteinizing hormone-releasing hormone (LHRH) test (**Table 1**), which revealed adrenal insufficiency, growth hormone deficiency, secondary hypogonadism, and secondary hypothyroidism. Panhypopituitarism was diagnosed. Water deprivation/desmopressin tests (**Table 1**) showed increased urine osmolarity and decreased urine amount post desmopressin (DDAVP) infusion. Central DI was confirmed.

**Fig. 1:**
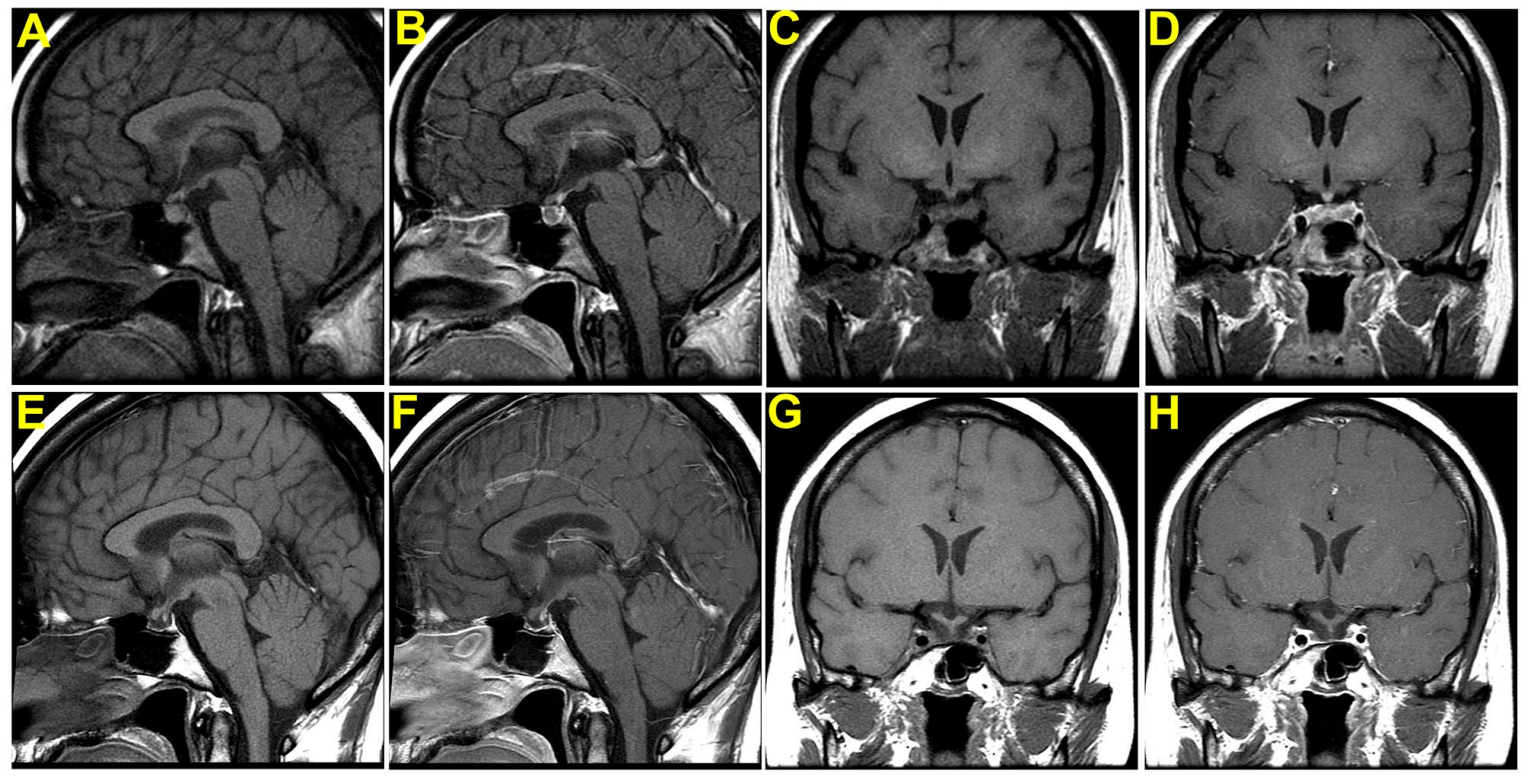
MRI for head with the focus in the sella with and without Gadolinium contrast medium. MRI for head with the focus in the sella, sagittal view without Gadolinium contrast medium (**A**), and with Gadolinium contrast medium (**B**), corneal view without Gadolinium contrast medium (**C**) and with Gadolinium contrast medium (**D**), revealed there was slight heterogeneous signal within the pituitary gland. The size of the pituitary gland was within normal range. There was no normal enhancement of the gland itself except the pituitary stalk. The picture of hemorrhage and necrosis of the pituitary gland was compatible with Sheehan’s syndrome. Follow-up MRI of sellar region after 10 years, sagittal view without Gadolinium contrast medium (**E**), and with Gadolinium contrast medium (**F**), corneal view without Gadolinium contrast medium (**G**) and with Gadolinium contrast medium (**H**) revealed very small pituitary gland. The previous intra-glandular hemorrhage was not visualized this time. The pituitary stalk was in the midline

Magnetic resonance imaging (MRI) of the sellar region (Fig. 1) was performed one month postpartum, revealing a heterogeneous signal within the normal-sized pituitary gland, consistent with acute hemorrhage and necrosis. A follow-up sellar region MRI (Fig. 1), at 10 years later, revealed a very small size of the remaining pituitary gland.

Following the diagnosis of Sheehan’s syndrome with DI, the patient received intranasal desmopressin acetate 10 mcg BID, oral cortisone acetate 25 mg BID, levothyroxine sodium 100 mcg QD, and conjugated estrogens 0.625 mg QD to address the hormonal deficiencies. Over ten years of follow-up, her panhypopituitarism persisted, necessitating long-term hormone replacement therapy.

## Discussion

Sheehan’s syndrome is classically characterized by reduced anterior pituitary hormone secretion following postpartum shock or hemorrhage. In 1963, Sheehan and Whitehead described varying degrees of pathological alterations including atrophy and scarring change in 90% of the posterior pituitary lobes from postpartum hypopituitary patients examined post mortem. Whitehead also reported constant hypothalamic damage characterized by marked atrophy of the supraoptic nuclei and, to a lesser extent, of the paraventricular nuclei, though, none of these patients had a history of transient DI [1].

Over the years, several studies had investigated antidiuretic function in patients with Sheehan’s syndrome [3–8]. Despite being asymptomatic for polyuria, most patients showed impaired osmoregulation of vasopressin secretion, including impaired maximal urine osmolality, insufficient urinary arginine vasopressin (AVP) excretion, elevated plasma osmolality, reduced urine-plasma osmolality ratios, and impaired plasma vasopressin response to plasma osmolality compared with normal control groups, both at baseline or after provocative test such as water deprivation / vasopressin or hypertonic saline infusion test. Patients also took a longer time to reach maximal urinary osmolality compared with normal subjects during functional tests.

From a clinical perspective, DI associated with Sheehan’s syndrome exhibits characteristic features. First, it tends to be partial rather than complete DI. Bakiri et al. and Jialal et al. reported that patients diagnosed with Sheehan’s syndrome and DI had a less than 50% increase in their maximal urine osmolality after water deprivation/vasopressin test [4–6]. Therefore, they concluded that failure of arginine-vasopressin secretion is more often partial. Atmaca, Tanriverdi et al. also disclosed that the patients diagnosed with DI all revealed partial DI. Hence, long term treatment of DI may be necessary in only a few cases.

Second, the onset of DI in Sheehan syndrome tends to be late, and the course tends to be transient. In Iwasaki’s series, only 4 of 12 had a history of transient symptomatic polyuria in their immediate postpartum period and one had DI for about 7 months after delivery. None of them developed permanent DI (all remission 10 months later). Robert L. at el. reported a case report with DI and Sheehan syndrome, and the interval between the postpartum hemorrhage and symptoms of polyuria was 11 years [9].

The reasons for DI to be partial and transient might be explained. The function of some vasopressin neurons that had been damaged as a result of the pituitary ischemic may recover. In addition, a decrease in the metabolism of vasopressin caused by disappearance of plasma vasopressinase that normally circulates during pregnancy also contributes. This enzyme is known to persist for 4–6 weeks after delivery. Finally, chronic deficiency of the hormone may lead to increased renal sensitivity to vasopressin.

Our case was diagnosed with adrenal insufficiency, which presented persistent fever on the 25th day after postpartum hemorrhage. Polyuria occurred soon after glucocorticoid replacement on the 30th day. Due to glucocorticoid deficiency, it is reasonable to presume that her DI might onset earlier than we noticed. After a water deprivation/desmopressin test, central DI, characterized by decreased release of AVP, was diagnosed. She was followed for years till now, and DDAVP therapy was still necessary for her to relieve polyuria. In comparison with the previously reported cases, the DI in this patient was unique as it occurred after steroid replacement for adrenal insufficiency, resulted in complete loss of antidiuretic function, had an early-onset, and was permanent in course.

Interestingly, although early research mostly suggested that DI was late onset, the fact is that early onset is not rare. Olmes, Solomayer et al. had performed a systematic review of Sheehan’s syndrome and DI, and identified eight relevant case reports published between 1990 and 2021 [10]. Most of the patients encountered severe blood loss, following hypotension or shock, similar to our case. The interval between birth and the initial manifestation of symptoms ranged from 24 h to 19 days after delivery in all cases [2, 10–17]., with a median of 8.8 days. In our case, the patient developed symptoms on the 30th day. Five reports described accompanying hormone disorders with adrenal insufficiency, hypothyroidism and hypogonadism. Moreover, six reports mentioned the diagnosis of central DI, and the use of desmopressin to treat DI in the context of Sheehan’s syndrome.

The possible mechanism by which glucocorticoid replacement exacerbates DI is that glucocorticoids inhibit the synthesis of AVP in the hypothalamus through negative feedback. In return, hypocortisolism can result in elevated AVP levels and increased sensitivity to AVP [2, 18, 19]. Therefore, in our case, hypocortisolism might have initially masked the clinical manifestations of AVP deficiency; however, once steroid replacement therapy was initiated, the deficiency in AVP became symptomatic.

As for the pathogenesis of neurohypophyseal damage, it is difficult to determine. During pregnancy, the pituitary gland is enlarged physiologically [20]. Pituitary enlargement causes compression of the anterior hypophyseal artery, resulting in mild pituitary ischemia. Severe postpartum hemorrhage may lead to necrosis of the enlarged pituitary glands [12]. Typically, Sheehan’s syndrome presents initially as anterior pituitary hormonal dysfunction. Although most patients with Sheehan’s syndrome had atrophy of the posterior pituitary lobe and hypothalamic nuclei, AVP deficiency manifested as DI is very rare and occurs when a large portion of the neurohypophysis is destroyed [1]. However, it is still unclear whether DI is due to the posterior pituitary lobe damage or the insufficient hormone secretion by the hypothalamus.

Imaging study may offer an insight into such an issue. MRI imaging in our patient at the time of diagnosis revealed hemorrhage and necrosis of the pituitary gland, a distinct feature from previously reported ones. Originally, Sheehan’s syndrome was described as ischemic necrosis pathologically in the anterior pituitary following postpartum shock or hemorrhage without any image characteristics mentioned. However, depending on the time interval of event and image taking, non-hemorrhagic pituitary apoplexy [21] or empty sella have been the most common image finding in Sheehan’s syndrome. There were only few reports like ours on early image findings after postpartum hemorrhage in Sheehan’s syndrome. In a study measuring sella size and content in Sheehan’s syndrome by high resolution computerized tomography with time interval from postpartum hemorrhage to scan varied from 0.5 to 22 years (mean 11 years), an empty sella of a relatively small size was reported to be a common radiological finding, and the content varies from pituitary stalk only (26/57), stalk and other pituitary tissues (20/57), and a sella of pure CSF density (11/57) [22]. The hemorrhage of pituitary gland initially in our case might be due to hemorrhagic infarction or bleeding tendency because of complicated DIC course.

The extent of neurohypophyseal and adenohypophyseal defects was reported to be correlated. Iwasaki found 4 patients with very poor vasopressin response to hypertonic saline infusion had almost complete anterior pituitary hormone deficiency, where 4 other patients with normal or nearly normal vasopressin response had only partial or isolated anterior pituitary hormone deficiency [3]. Therefore, the risk for development of posterior pituitary dysfunction is increased if more complete loss of anterior pituitary hormone function loss exists [23]. It was applied to our case. Our patient suffered from anterior hypopituitarism with concurrent DI.

## Conclusion

Sheehan syndrome is a rare complication of postpartum hemorrhage. Central diabetes insipidus should be suspected, although not commonly, while the patient presented polyuria and polydipsia. Besides, the potential necessity for long-term hormonal replacement therapy should be considered.

**Table 1:** Functional Tests to evaluate anterior pituitary function and to diagnosis of diabetes insipidus

Insulin hypoglycemia test/ TRH test/LHRH Test	0 min		30 min		45 min			60 min		75 min			90 min		
Plasma glucose (mg/dL)	79		44		55			63		67			70		
Cortisol (µg/dL)	2.0		1.9		1.6			1.6		1.7			1.6		
Growth hormone (ng/mL)	< 0.05		< 0.05		< 0.05			< 0.05		< 0.05			< 0.05		
FSH (mIU/mL)	0.9		0.9					0.9					0.9		
LH (mIU/mL)			0.78					0.78					0.79		
PRL (ng/mL)	1.2		1.3		1.2			1.1							
TSH(µU/mL)	0.31		0.49		0.41			0.39					0.32		
T4 (µg/dL)	1.6														
T3 (ng/dL)	64.5														
**Water deprivation/ desmopressin test**	**-6 h**	**-5 h**		**-4 h**		**-3 h**	**-2 h**		**-1 h**		**0hr** ^**a**^	**1 h**		**2 h**	
BP (mmHg)	136/100	110/86		132/90		130/94	138/98		136/100		134/100	138/90		120/86	
PR(/min)	96	88		86		82	82		86		88	86		88	
BW(Kg)	76	75.5		75.5		75.2	73		74.5		74	74		74	
Urine osmolarity	116	137		153		158	165		189		206	372		411	
Plasma osmolarity	300	298		311		307	313		315		310	-		-	
Urine amount	-	260		270		350	390		380		250	170		50	

a. DDAVP 1 mg was administered at 16:00. (0hr)

## Data Availability

No datasets were generated or analysed during the current study.

## Abbreviations

DI: diabetes insipidus
RBC: red blood cells
FFP: fresh frozen plasma
PCO_2_: partial pressure of carbon dioxide
PO_2_: oxygen partial pressure
DDAVP: desmopressin
TRH: thyrotropin-releasing hormone
LHRH: luteinizing hormone releasing hormone
MRI: magnetic resonance imaging
BID: twice daily
QD: every day
AVP: arginine vasopressin
